# Influence of Acute Jugular Vein Compression on the Cerebral Blood Flow Velocity, Pial Artery Pulsation and Width of Subarachnoid Space in Humans

**DOI:** 10.1371/journal.pone.0048245

**Published:** 2012-10-24

**Authors:** Andrzej F. Frydrychowski, Pawel J. Winklewski, Wojciech Guminski

**Affiliations:** 1 Institute of Human Physiology, Medical University of Gdansk, Gdansk, Poland; 2 Department of Computer Communications, Gdansk University of Technology, Gdansk, Poland; Charité University Medicine Berlin, Germany

## Abstract

**Purpose:**

The aim of this study was to assess the effect of acute bilateral jugular vein compression on: (1) pial artery pulsation (cc-TQ); (2) cerebral blood flow velocity (CBFV); (3) peripheral blood pressure; and (4) possible relations between mentioned parameters.

**Methods:**

Experiments were performed on a group of 32 healthy 19–30 years old male subjects. cc-TQ and the subarachnoid width (sas-TQ) were measured using near-infrared transillumination/backscattering sounding (NIR-T/BSS), CBFV in the left anterior cerebral artery using transcranial Doppler, blood pressure was measured using Finapres, while end-tidal CO_2_ was measured using medical gas analyser. Bilateral jugular vein compression was achieved with the use of a sphygmomanometer held on the neck of the participant and pumped at the pressure of 40 mmHg, and was performed in the bend-over (BOPT) and swayed to the back (initial) position.

**Results:**

In the first group (n = 10) during BOPT, sas-TQ and pulse pressure (PP) decreased (−17.6% and −17.9%, respectively) and CBFV increased (+35.0%), while cc-TQ did not change (+1.91%). In the second group, in the initial position (n = 22) cc-TQ and CBFV increased (106.6% and 20.1%, respectively), while sas-TQ and PP decreases were not statistically significant (−15.5% and −9.0%, respectively). End-tidal CO_2_ remained stable during BOPT and venous compression in both groups. Significant interdependence between changes in cc-TQ and PP after bilateral jugular vein compression in the initial position was found (r = −0.74).

**Conclusions:**

Acute bilateral jugular venous insufficiency leads to hyperkinetic cerebral circulation characterised by augmented pial artery pulsation and CBFV and direct transmission of PP into the brain microcirculation. The Windkessel effect with impaired jugular outflow and more likely increased intracranial pressure is described. This study clarifies the potential mechanism linking jugular outflow insufficiency with arterial small vessel cerebral disease.

## Introduction

A stable increase in cerebral venous pressure induces arteriolar vasodilation, which averages 6% to 12% of the control diameter in cats [1], [2] and dogs [3]. Acute superior vena cava occlusion results in increased pial venous pressure and subsequent blood-brain barrier disruption in rats [4]. Unilateral internal and external jugular vein ligation in swine increases bihemispheric cerebral blood flow and metabolism [5], and progressive superior vena cava obstruction produces measurable signs of impaired cerebral perfusion in pigs [6]. As cranial window installation and microscopic examination remain the methods of choice to investigate the pial microvessels in vivo [7], for obvious reasons, the effect of increased cerebral venous pressure has not yet been investigated in humans.

The amplitude of pial artery pulsation (cc-TQ) can be measured non-invasively using near-infrared transillumination/backscattering sounding (NIR-T/BSS), a new method based on infrared radiation (IR) that has been developed in the last decade by our team [8]–[12]. Contrary to near-infrared spectroscopy (NIRS), which relies on absorption of IR by haemoglobin [13], [14], NIR-T/BSS uses the subarachnoid space (SAS) filled with translucent cerebrospinal fluid (CSF) as a propagation duct for IR. We have previously shown that pial artery pulsation may serve as a sensitive index of changes in microvessel compliance. cc-TQ increases were observed during acetazolamide and hypercapnic tests, acute hypoxia, papaverine and glucagon administration and electroconvulsive therapy, while cc-TQ decreases were recorded during the stabilisation period after the abovementioned procedures [10], [15]–[17]. Significant cc-TQ decrease was seen during handgrip test [18]. In addition, NIR-T/BSS allows for assessment of changes in the width of SAS [19], indicative in changes in CSF volume and intracranial pressure [8], [10]–[12], [20]. Due to non-invasiveness, ease of use and low cost NIR-T/BSS potentially constitutes an ideal tool to monitor brain microcirculation over long periods of time.

We hypothesised that impaired venous outflow would lead to pial artery dilation caused by a metabolic and/or autoregulatory mechanism due to decreased oxygen supply and/or increased intracranial pressure, respectively. Augmented pial artery compliance should result in increased cc-TQ and hyperdynamic brain circulation characterised by augmented cerebral blood flow velocity (CBFV). The investigated topic is not a purely theoretical concept, as increased cerebral venous blood volume has been recently reported during sympathetic activation (for a review, see [21]). Therefore, the brain maybe exposed to venous congestion much more frequently than initially thought. Furthermore, venous dysfunction is increasingly linked to pulse wave encephalopathy and white matter changes [22]–[25]. The aim of this study was to assess in healthy volunteers the effect of acute jugular venous compression on: (1) cc-TQ; (2) CBFV; (3) peripheral blood pressure (BP); and possible relations between CBFV, BP and cc-TQ.

## Materials and Methods

### Subjects

Experiments were performed on a group of 32 healthy 19–30 year old male subjects. The volunteers were selected on the basis of a medical questionnaire, interview and blood pressure measurement. All volunteers received detailed information about study objectives and potential adverse reactions (transient headache, vertigo and blood flushes) and gave written informed consent to participate in the study. The experimental protocol and the study were approved by the ethical committee of the Medical University of Gdansk (TKEBN/259). Subjects did not have any disorders and were not taking any medication. Before the experiment, each subject underwent a general and neurological examination. No nicotine, coffee, tea, cocoa or any methylxanthine-containing food or beverages were permitted for 8 hours before the tests. Additionally, prior to each test, the volunteers were asked to sit comfortably and rest for 30 minutes.

### Experimental design

#### Bend Over Position Test (BOPT)

- Initial position: subject set comfortably for 10 minutes with head swayed to the back at 20° angle.
- BOPT: subject bends forwards at 45° angle (the trunk and the head) from the initial half-lying position on the back [10], [18].

Then, for bilateral jugular vein compression participants were divided into two groups.

#### First group consisting of 10 subjects

- Jugular veins were completely compressed 3 minutes after starting BOPT for 3 minutes.
- Subject returned to initial position.

#### Second group consisting of 22 subjects

- Subject returned to initial position 3 minutes after starting BOPT.
- Subject set comfortably in initial position for 3 minutes.
- Jugular veins were completely compressed for 3 minutes.

The numerical values for statistical analysis were taken just before the end of each three-minute procedure. After bilateral jugular vein compression all subject were asked to sit comfortably in the initial position for 10 minutes.

Bilateral jugular vein compression was achieved with the use of a sphygmomanometer held on the neck of the participant and pumped to a pressure of 40 mmHg. Blood pressure in jugular veins in the initial position should be close to zero, or even negative, so it can be assumed that compression was complete. The subjects were asked to breathe normally to avoid changes in PaCO_2_.

### Transcranial Doppler measurement

Transcranial Doppler (TCD) measurement of cerebral blood flow velocity (CBFV) in the left anterior cerebral artery was performed with a Doppler ultrasound device TDS 4 (Sonomed, Warsaw, Poland). A pulse probe of 2 MHz was used and analysis of the results was carried out on a built-in IBM PC computer. To assure the best possible reproducibility of the recordings, the Doppler probe was mounted on the head with a special stabilising strip.

### Blood pressure measurement

Recording of changes in systolic arterial pressure (SAP), diastolic arterial pressure (DAP) and HR were measured using Finapres (Finapres, Ohmeda, Englewood, CO, USA). The Finapres sensor was mounted onto the middle finger of the non-dominant hand resting on the table. Beat-to-beat blood pressure was transferred to a computer console continuously displaying SAP, DAP and HR. Pulse pressure (PP) was calculated from the following equation: PP = SAP−DAP.

### End-tidal CO_2_ measurement

End-tidal CO_2_ was measured in mmHg with a medical gas analyser (Datex Instrumentarium, Helsinki, Finland). The instrument was calibrated using a certified standard gas mixture before each experiment. End-tidal CO_2_ was recorded and printed continuously in real time. Values for subsequent analysis were read from the printouts and entered manually into a Statistics for Windows 8.0 database.

**Figure 1:**
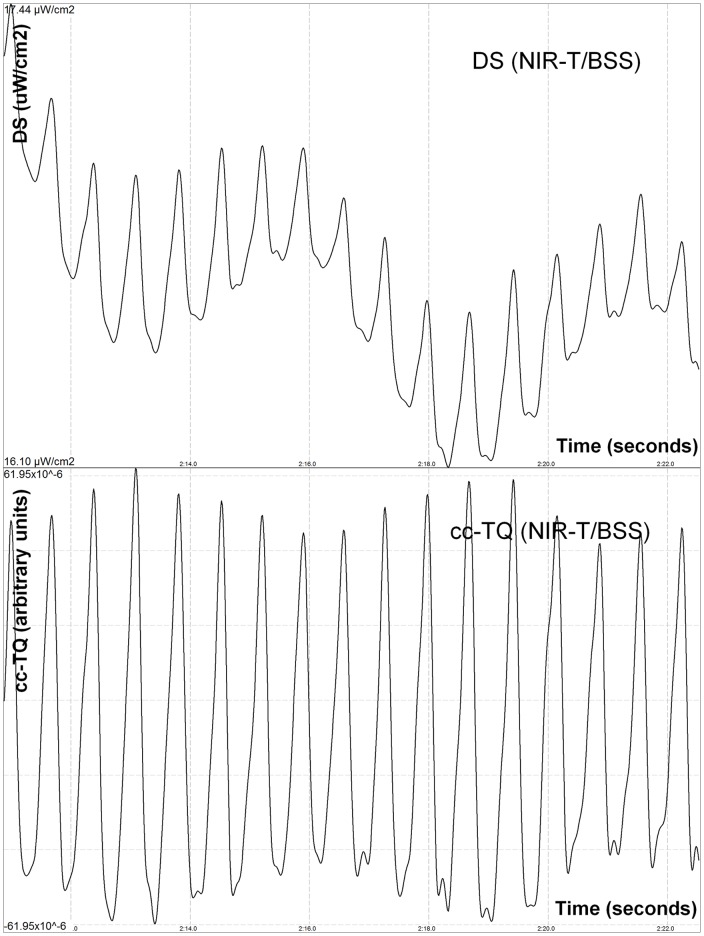
Effect of acute bilateral jugular vein compression on NIR-T/BSS variables during BOPT: cc-TQ is “cut” by the narrowing SAS. Sharp edges of the cc-TQ waves are visible at the distant sensor (DS) and cc-TQ (enhanced tracings). cc-TQ – cardiac component of transillumination quotient (pial artery pulsation); µW/cm^2^ – microwatt/centimetre^2^.

**Figure 2:**
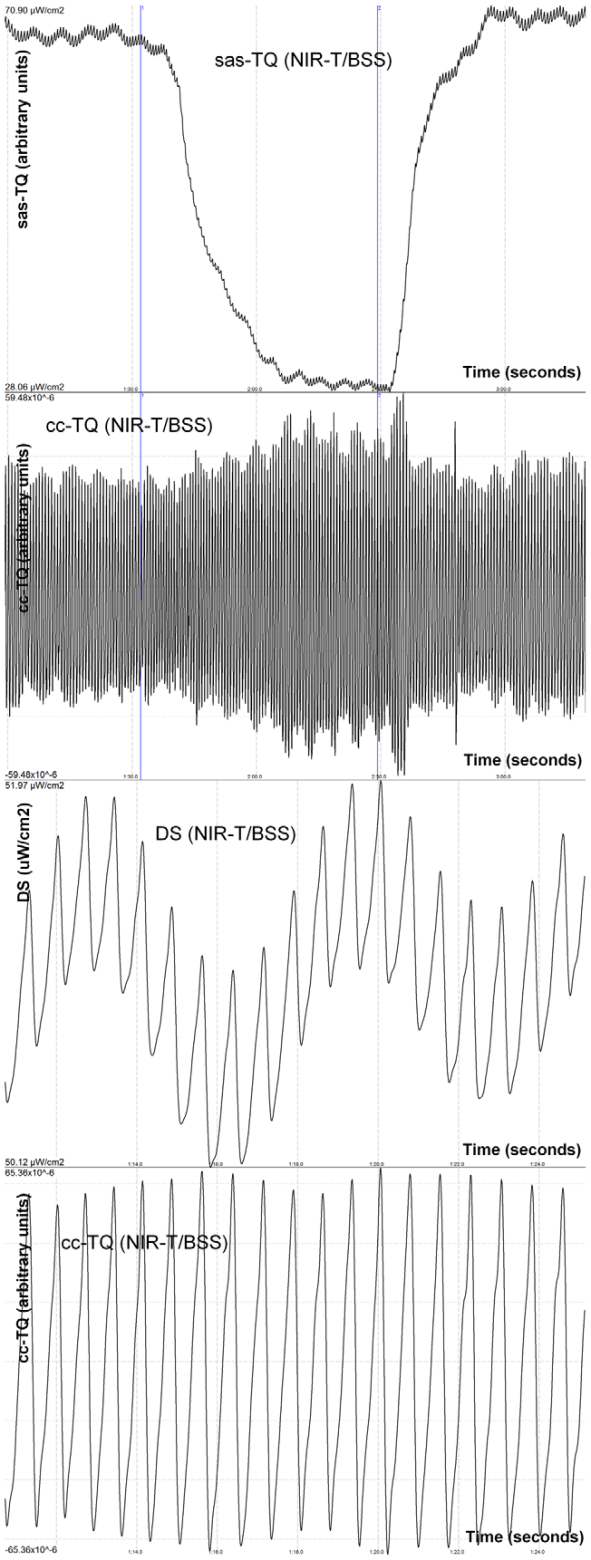
Effect of acute bilateral jugular vein compression on NIR-T/BSS variables in the initial position. 1 – sas-TQ, 2 – cc-TQ, 3 – enhanced tracing of the distant sensor (DS) signal (absence of the “cutting” effect and absent sharp edges), 4 – enhanced tracing of cc-TQ (absence of the “cutting” effect and absent sharp edges). sas-TQ – the subarachnoid component of the transillumination quotient (the subarachnoid width); cc-TQ – cardiac component of transillumination quotient (pial artery pulsation); µW/cm^2^ – microwatt/centimetre^2^.

**Table 1:** Effects of acute bilateral jugular vein compression on sas-TQ, cc-TQ, CBFV, SAP, DAP, PP and HR during the BOPT test (BOPT – JVO; n = 10). Mean values and Standard Deviations (SD) are provided.

	Initial position (baseline)	BOPT% Change		BOPT – JVO% Change		Initial position(recovery % Change	
sas-TQ (arbitrary units)	1623±55.7	1123±48.1***	−30,8	926±36.7***	−17.6	1178±50.8**	27.2
cc-TQ (arbitrary units)	54,5±24,2	34.0±12.8**	−37.5	34.7±17.0^NS^	1.91	33.3±11.3**	−3.95
CBFV (cm*sec^−1^)	59.3±14.1	45.1±9.7**	−24.0	60.9±11.6^**^	35.0	51.8±13.4^**^	−15.0
SAP (mmHg)	138.0±12.3	131.0±12.0**	−5.07	131.7±17.3^NS^	0.53	126.5±14.3**	−3.95
DAP (mmHg)	74.1±9.1	73.9±9.2^NS^	−0.27	84.8±12.4**	14.75	73.6±9.7^NS^	−13.2
PP (mmHg)	63.9±8.8	57.1±7.9**	−10.6	46.9±14.2**	−17.9	52.9±12.1**	12.8
HR (beats*sec^−1^)	75.6±7.9	78.3±6.4^NS^	3.57	83.5±9.6*	6.64	52.9±12.8**	−6.83
End-tidal CO_2_ (mmHg)	35.6±1.7	36.8±3.2^NS^	3.37	36.3±2.9^NS^	−1.4	36.1±5.3^NS^	−0.56

All changes are calculated versus preceding values. *p<0.05; **p<0.01; ***p<0.001. sas-TQ – the subarachnoid component of the transillumination quotient (the subarachnoid width); cc-TQ – cardiac component of transillumination quotient (pial artery pulsation); CBFV – cerebral blood flow velocity; SAP – systolic arterial pressure; DAP – diastolic arterial pressure; PP – pulse pressure; HR – heart rate.

**Table 2:** Effects of acute bilateral jugular vein compression on sas-TQ, cc-TQ, CBFV, SAP, DAP, PP and HR in initial position (Initial – JVO; n = 22).

	Initial position (baseline)	BOPT% Change		Initial position(recovery after BOPT)% Change		Initial – JVO % Change	
sas-TQ (arbitrary units)	1845±81.2	1287±61.8***	−30.2	1861±78.5***	44.6	1573±79.8^NS^	−15.5
cc-TQ (arbitrary units)	70.8±53.7	42.8±41.6*	−39.7	67.1±54.4*	56.8	138,6±32.8*	106.6
CBFV (cm*sec^−1^)	62.3±11.7	47.8±10.4**	−23.3	61.2±17.4**	28.0	73.5±15.4**	20.1
SAP (mmHg)	140.8±11.9	131.5±18.2**	−6.7	140.6±12.0^NS^	6.9	140.3±10.5^ NS^	−0.03
DAP (mmHg)	79.7±9.2	78.3±9.4^NS^	−1.7	79.4±8.7*	1.4	82.6±7.9^ NS^	4.0
PP (mmHg)	61.1±7.0	53.1±15.5*	−13.1	61.2±7.1**	15.3	57.7±5.0^NS^	−9.0
HR (beats*sec^−1^)	77.9±10.4	79.0±11.3^NS^	1.3	77.4±9.9^NS^	2.0	79.0±10.0*	2.1
End-tidal CO_2_ (mmHg)	37.2±4.3	36.4±3.1^NS^	−2.2	36.8±3.6^NS^	1.1	37.1±4.8^NS^	−0.8

Mean values and Standard Deviations (SD) are provided. All changes are calculated versus preceding values. *p<0.05; **p<0.01; ***p<0.001. sas-TQ – the subarachnoid component of the transillumination quotient (the subarachnoid width); cc-TQ – cardiac component of transillumination quotient (pial artery pulsation); CBFV – cerebral blood flow velocity; SAP – systolic arterial pressure; DAP – diastolic arterial pressure; PP – pulse pressure; HR – heart rate.

### NIR-T/BSS measurement

Recording of changes in the amplitude of pial artery pulsation and in the width of SAS with NIR-T/BSS were performed with a head-mounted SAS 100 Monitor (NIRT sp. z o.o., Wierzbice, Poland). The sensor unit consists of the emitter (E) and two photo-sensors located at different distances from the emitter. The NIR-T/BSS emitter is a near-infrared light-emitting diode (LED). The proximal sensor (PS) is located close to the emitter, while the distal sensor (DS) is located at a larger distance from the emitter. The stream of IR generated by the emitter penetrates the highly perfuse layer of the skin of the head, the skull bones and the SAS. The stream of radiation reflects from the surface of the brain and reaches the sensors, crossing the aforementioned layers of tissues in reverse order. Signals from the sensors undergo analogue-digital conversion in a specialised data acquisition system and are recorded on the microcomputer' s hard disk for subsequent analysis with on-line presentation on the computer monitor.

**Figure 3:**
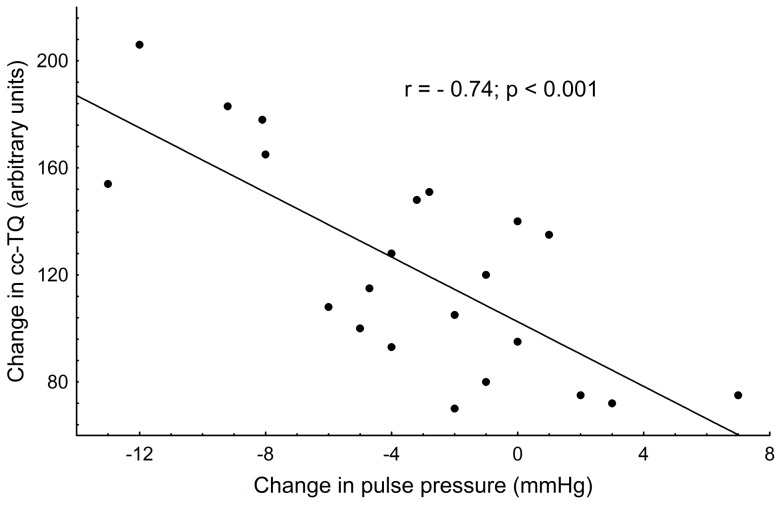
Linear correlation analysis of cc-TQ and PP change during bilateral jugular vein compression in initial position versus baseline. All values of cc-TQ and PP obtained from 22 volunteers were included in the analysis of linear correlation (Spearman r = −0.74, p<0.001). cc-TQ – cardiac component of transillumination quotient (pial artery pulsation); PP – pulse pressure; mmHg – millimetres of mercury.

Theoretical and practical foundations of the NIR-T/BSS method were provided in the model studies by our team [8], [10]–[12], [20]. Briefly, a signal received by the DS is divided by the signal received by the PS. Such division reduces proportional factors that affect each of the two signals in an identical way, because the quotient of these factors assumes the value 1. Both the dividend, i.e., the power of the DS signal, and the divisor, i.e., the power of the PS signal, are influenced by the width of SAS, and also by any factor capable of changing that width. Therefore, the quotient of the two signals, called the transillumination quotient (TQ), is sensitive to changes in the width of the SAS. The oscillations of TQ have their origin in different modulation of the PS and DS signals, namely in the modulation of the DS signal on its way through the SAS. It happens because only the DS receives radiation propagated within the SAS. Propagation of IR in the skin and bone is much worse than in the clear translucent cerebrospinal fluid (CSF) contained in the SAS, and with the DS placed far enough from the emitter, no radiation propagated in the superficial tissue layers can reach the DS [8], [10]–[12], [20]. The power of the IR stream reaching the DS is directly proportional to the width of the SAS. The wider the SAS, or the propagation duct, the more radiation reaches the DS and the greater the signal from that sensor, which is the dividend in the calculation of the TQ [8], [10]–[12], [20]. Conversely, the power of the IR stream reaching the PS is inversely proportional to the width of the SAS. Thus, the wider the SAS, the longer the distance between the inner skull bone surface and the brain at which IR energy is dissipated, and therefore the less IR follows the return route to the PS. The narrower the SAS, the closer the reflecting surface of the brain is to the PS and the more IR reaches that sensor.

Thus, in the transillumination quotient (TQ) we can identify three main components:

1. constant or a non-pulsatile component, further referred to as the subarachnoid component (sas-TQ); its value depending on the width of the CSF-filled SAS,
2. slow-variable pulsation, further referred to as the subcardiac component (scc-TQ); including, but not limited to, pulsation of a respiratory origin,
3. fast-variable pulsation, further referred to as the cardiac component (cc-TQ); resulting from heart-generated arterial pulsation that is the cause of fast oscillations of the width of SAS.

For further analysis, the first harmonic of the arterial pulsation-dependent oscillations of TQ was extracted through appropriate filtering, along with its modulation. Modulation of that harmonic is a fast-variable component (or cardiac component) of principal, second, and third harmonics of cardiac component waveform, respectively.

### Statistical analysis

W Shapiro-Wilk W, Mann-Whitney U and ANOVA tests were used to analyse the differences between average values. Changes in sas-TQ, cc-TQ, CBFV, SAP, DAP, PP, HR and end-tidal CO_2_ responses were compared to baseline values. Correlation and regression analysis was performed to assess interdependences between SAP, DAP, PP, HR, CBFV, end-tidal CO_2_, sas-TQ and cc-TQ. All statistical calculations were performed using the Statistics for Windows 8.0 commercial package.

## Results

Both groups (acute jugular vein compression in BOPT and initial position) were well-matched at baseline in terms of CBFV and BP. The BOPT test caused sas-TQ, cc-TQ, CBFV and PP decrease in both investigated groups (n = 10 and n = 22). However, acute bilateral jugular vein compression brought different results between the investigated groups. In the first group (n = 10) during BOPT sas-TQ and PP further decreased, CBFV increased while cc-TQ did not change (Fig. 1). In the second group, after return to initial position, during bilateral jugular vein compression (n = 22) cc-TQ and CBFV increased, while sas-TQ and PP decrease were not statistically significant (Fig. 2). End-tidal CO_2_ remained stable during BOPT and venous compression in both groups. Detailed descriptive statistics are provided in Table 1 and Table 2.

Correlation and regression analysis revealed significant interdependence between changes in cc-TQ and PP after bilateral jugular vein compression in the initial position (r = −0.74; p<0.001; Fig. 3).

## Discussion

This study, to the best of our knowledge, describes the effects of venous congestion on the pial arteries and CBFV in humans for the first time, confirming the results of earlier animal studies [1]–[3]. The new findings are that acute bilateral jugular outflow congestion in humans leads to hyperkinetic cerebral circulation, increased pial artery pulsation and direct transmission of peripheral PP into pial arteries. It is unlikely that the hyperdynamic brain circulation was caused by increased PaCO_2_ since the changes in end-tidal CO_2_ were not clinically relevant. Therefore, venous congestion is independent of hypercapnia in the induction of increased cerebral blood flow. Thus, the current study contributes to a better understanding of the mechanisms underlying the pathology of diseases characterised by increased cerebral venous blood volume.

The brain is subject to gravitation and changes its position along with changes in the position of the head. At a forward bending position (during BOPT) or in the abdominal-lying position, the brain floats forward toward the inner surface of the frontal bones and the SAS in the frontal region assumes its minimum value [19]. The observed decreases in sas-TQ and cc-TQ during the BOPT have already been discussed in detail [10], [18], [19]. What is important, and has not been presented earlier, is that decreases in cc-TQ are fully consistent with CBFV decreases observed in TCD. Hayreh and Edwards [26] demonstrated that during the very early phase of intracranial pressure elevation PP may decrease. Importantly, Wein and Kontos [2] reported a small but consistent decrease in BP after the onset of venous hypertension. Thus, the observed decrease in PP may suggest a slight increase in intracranial pressure during BOPT. However, based on the NIR-T/BSS model, we are not able to distinguish if a decrease in sas-TQ is solely due to physical brain movement or is in addition influenced by increased intracranial pressure.

Bilateral jugular vein compression resulted in enhanced CBFV in both groups. We may assume that hampered jugular outflow results in decreased oxygen supply, which in turn lead to arteriole dilation and subsequent increase in cerebral blood flow [5]. Pial artery dilation and increased compliance results in higher amplitude of pial artery pulsation [10], [15]–[17]. Alternatively, bilateral jugular vein compression more likely evoked an increase in intracranial pressure. Even a mild increase in intracranial pressure results in an autoregulatory response to diminished cerebral perfusion pressure, i.e. pial artery dilation and increased pulsation [10], [12], [20]. Therefore, cc-TQ should have been increased too. However, when jugular veins were compressed during BOPT, cc-TQ did not change. To explain this result we need to focus on assumptions built in the NIR-T/BSS model. NIR-T/BSS uses the SAS filled with CSF as a propagation duct for IR. Therefore the presence of SAS is the sine qua non condition for measurements of any NIR-T/BSS parameters. The SAS already decreased during BOPT gets even more squeezed during acute bilateral jugular vein compression, due to expanded intracranial blood volume, and more likely increased intracranial pressure. Therefore, the “cutting” effect actually develops due to the Windkessel mechanism. In order to compensate for higher resistance in the venous drainage pathways, the pial artery tries to dilate. However, this is not possible because the SAS is compressed and non-compliant. What we can see in Fig. 1 is that the amplitude of CVP is being cut by the narrowing SAS. This is an extremely important feature of NIR-T/BSS as the “cutting” of pial artery pulsation can be indicative of early brain oedema in human (unpublished results from our lab). BOPT, which was developed by our team 10 years ago, can be used to non-invasively assess brain reserve volume. Nevertheless, the above effect does not allow for assessment of acute bilateral vein compression influence on cc-TQ during BOPT. The “cutting” effect was observed in all volunteers during BOPT (n = 10). As the aim of this study was to assess pial artery pulsation during acute bilateral vein compression, we decided not to enrol more subjects into this experimental group.

Acute cerebral venous congestion produced significant cc-TQ increase in the initial position. Such a result is in line with the above-presented reasoning. In the initial position the SAS was insignificantly reduced by enhanced intracranial blood volume, and remained wide enough to allow for proper registration of the amplitude of pial artery pulsation (Fig. 2). Increases in CBFV and pial artery pulsation observed during acute impairment of venous outflow are consistent with the model proposed by Bateman [23]. Pulsatile flow is a manifestation of the energy stored in the form of the pulse pressure and, to ensure non-pulsatile continuous flow through the capillary bed, must be dampened by shifting cerebrospinal fluid and venous blood [27]. When venous outflow is impaired and intracranial pressure is elevated, the pulse pressure cannot be dampened and results in increased pial artery pulsation [28]. Correlation and regression analysis revealed strong interdependence between cc-TQ and PP during acute bilateral jugular vein compression in the initial position (Fig. 3). Exposure to highly pulsatile pressure and augmented flow is a known predictor of cerebral vascular damage, even in the absence of increases in mean BP [29]–[31]. We have already proved in an animal model that during hypercapnia pulsatile flow is directly transmitted into pial arteries [16]. The presented correlation may actually provide a link between cerebral venous insufficiency and cerebral arterial small vessel disease in human. Chronic cerebral insufficiency is associated with impaired cerebral perfusion [6], [32]. Toveda *et* *al*. [6] and Zamboni *et* *al*. [32] reports are not contradictory to our results. The Windkessel mechanism requires the SAS to be compliant to ensure smooth blood flow through the cerebral vascular bed. If the pial arteries cannot expand, then the flow through the vascular bed becomes more pulsatile and the shear forces on the arterial wall increase [23]. Direct transmission of PP into the brain microcirculation, if maintained over a longer period of time, may result in subsequent arteriole remodelling, microbleeding and increased vascular resistance, leading to an augmented vulnerability to ischaemia as the final outcome [29]–[31]. Furthermore, elevated arterial inflow combined with impaired venous outflow may lead to the development of normal pressure hydrocephalus [23], a disease associated with leukoaraiosis [22], [24], [25], altered cerebral pulsation propagation [22] and reduced cerebral blood flow [33]. Zamboni reported [32] a link between chronic cerebrospinal venous insufficiency and hypoperfusion in multiple sclerosis; although this remains controversial, it is in line with the reasoning presented above.

The following study limitations should be taken into account. Only young, healthy males were investigated and acute jugular vein insufficiency was analysed. Therefore, any extrapolations to patients suffering from chronic cerebral venous insufficiency or normal pressure hydrocephalus should be viewed with caution. In our study the SAS decrease during bilateral jugular vein compression in the initial position was not statistically significant, which may suggest that even during bilateral jugular vein congestion the brain may be decompressed through the vertebral plexus [34] or venous collateral circulation [35]. The high within- and between-subject reproducibility and repeatability of NIR-T/BSS measurements have been demonstrated earlier [9], [10], [19], [36]. NIR-T/BSS, like NIRS, allows for direct within-subject comparisons [10], [37]. Both groups (acute jugular vein compression in BOPT and initial position) were well-matched at baseline in terms of CBFV and BP. The observed differences in sas-TQ and cc-TQ at baseline, although not statistically significant, might have been related to differences in skull bone parameters. However, it should be noted that the percentage changes during BOPT were almost identical in both groups. This is in agreement with our previous studies showing that as long as changes from baseline values are analysed, high between-subject reproducibility is observed [10], [16]–[19], [36]. So far, measurements using IR light (NIRS and NIR-T/BSS) do not allow for direct between-subjects comparisons due to differences in skull bone parameters [10], [37].

In summary, acute bilateral jugular venous insufficiency leads to hyperkinetic cerebral circulation characterised by augmented pial artery pulsation and CBFV and direct transmission of PP into the brain microcirculation. From the mechanistic point of view, the Windkessel effect with impaired jugular outflow and most likely increased intracranial pressure is described. Impaired jugular outflow does not allow for dampening the pulsation energy and actually exaggerates the pulsatile flow, which in turn creates a hazardous environment for the brain microcirculation. Therefore, this study clarifies the potential mechanism linking jugular outflow insufficiency with small vessel arterial cerebral disease.
